# Complete transmural migration of a retained surgical sponge: an atypical case in image mimicking intussusception: A case report

**DOI:** 10.1097/MD.0000000000008246

**Published:** 2017-10-20

**Authors:** Yu Zhou, Ping Chen, Tang Qiao, Yi-feng Chen, Liang Zong

**Affiliations:** a Department of General Surgery, Suzhou Municipal Hospital (North Campus), Suzhou; b Department of Gastrointestinal Surgery, Clinical Medical College of Yangzhou University (the Northern Jiangsu People's Hospital), Yangzhou; c Medical Research Center, Clinical Medical College of Yangzhou University (the Northern Jiangsu People's Hospital), Yangzhou, Jiangsu Province, China.

**Keywords:** abdomen, intussusception, retained surgical sponge, transmural migration

## Abstract

**Rationale:**

Intraluminal migration of a retained surgical sponge causing intestinal obstruction and fistula is extremely rare occurrence.

**Patient concerns:**

A case of a 35-year-old male, who complaining a diffuse abdominal pain beginning three days earlier. He also complained of occasional vomiting, nonspecific abdominal pain, and an unintentional 15 kg weight loss during the past 2 years. However, there were no clear findings in previous laboratory work. He had received an open appendectomy approximately 4 years earlier.

**Diagnoses:**

Retained surgical sponge.

**Interventions:**

A contrast-enhanced CT of the abdomen showed a clear invagination of the small intestine. However, intraoperatively, we could not find an intestinal segment with intussusception. After the adhesive intestine was detached, a jejunal−ileal cross-linked fistula was found. More surprisingly, a retained surgical sponge was found inside the ileal fistula when the cross-linked fistula was detached.

**Outcomes:**

The patient was discharged 7 days after surgery.

**Lessons:**

This is the first report showing an atypical image of a complete transmural migration of a retained surgical sponge mimicking intussusception.

## 1 Introduction

Retained surgical sponge (RSS), usually referred to as gossypiboma (GP), is an avoidable complication, which, unfortunately, still occurs. Declaration is not done exactly due to legal-medical issues connected with this problem; a recent study estimated the incidence of retained surgical foreign bodies as 1 in 5500 operations. Patients with RSS can remain with unspecific clinical symptoms both in the early postoperative period and for months or years after the initial operation. Rarely, RSS is able to do intraluminal migration without creating any defect into the gastrointestinal tract. Diagnosis tends to be difficult. Abdominal x-ray, ultrasonography (US), computed tomography (CT), magnetic resonance imaging (MRI), and upper-gastrointestinal endoscopy can all be used to assist in diagnosis. In the present case, we aimed to report a case of an RSS, who survived 4 years after an appendix section, causing an intestinal obstruction due to its complete migration into the interior of the ileum and a fistula between jejunum and ileum, without any apparent opening in the intestinal wall.

This study was approved by the ethics committee of Clinical Medical College of Yangzhou University (Subei People's Hospital of Jiangsu Province), Yangzhou, China. The patient consented to the publication of this study.

## 2 Case report

**Figure 1:**
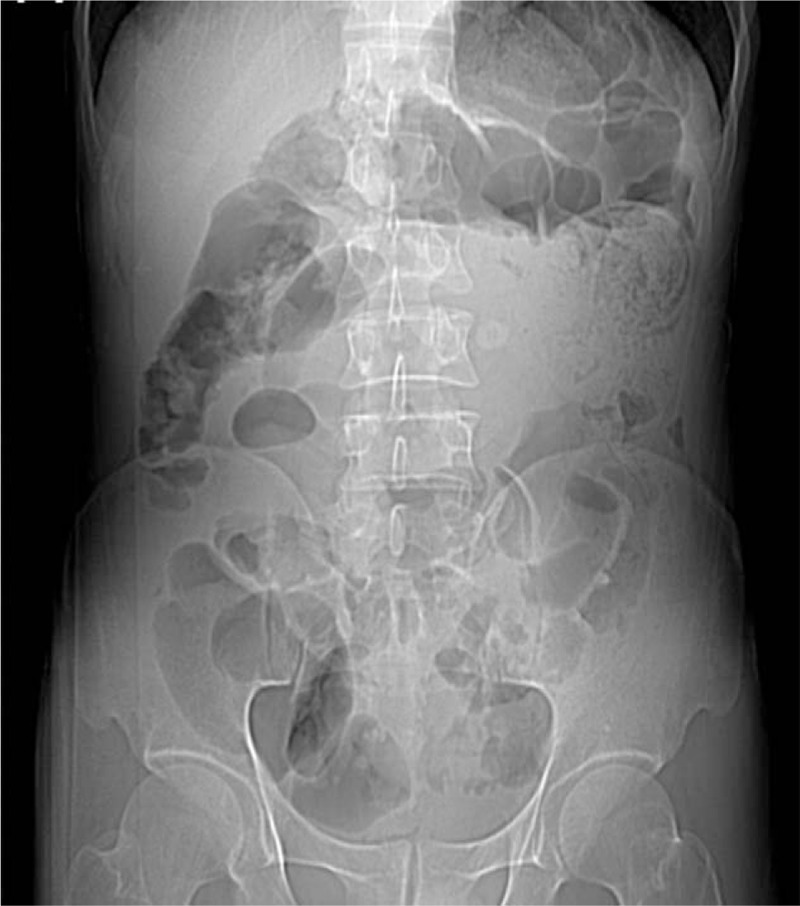
A contrast-enhanced CT of the abdomen showed a clear invagination of the small intestine.

**Figure 2:**
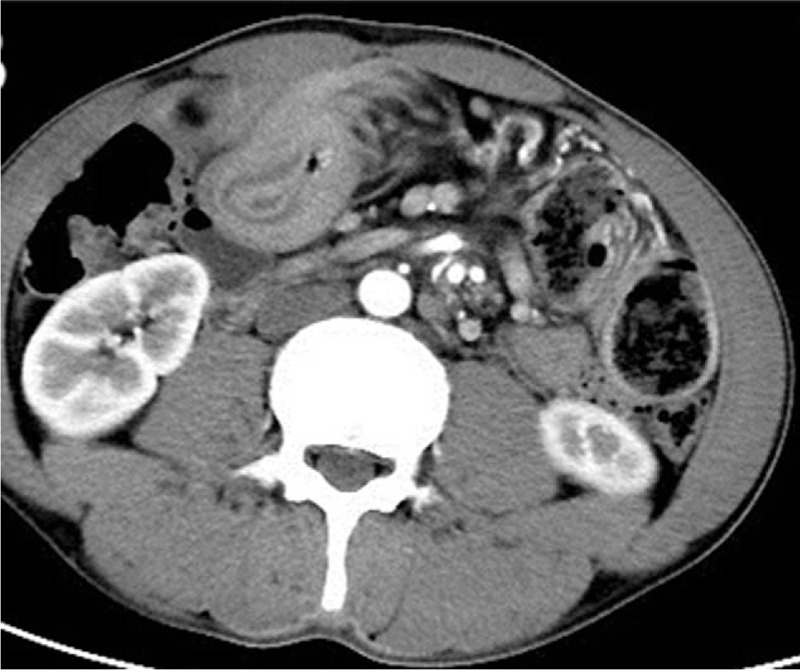
A contrast-enhanced CT of the abdomen showed a clear invagination of the small intestine.

A 35-year-old Chinese man was admitted to the hospital with a chief complaint of diffuse abdominal pain beginning 3 days earlier. On admission he was febrile, with a temperature of 38.6°C. He denied recent use of antibiotics. The patient also complained of occasional vomiting, nonspecific abdominal pain, and an unintentional 15 kg weight loss during the past 2 years. However, there were no clear findings in previous laboratory work, including a general blood test and a tumor markers test, or abdominal imaging. He had received an open appendectomy approximately 4 years earlier. Physical examination revealed diffuse tenderness in the epigastric region. Laboratory tests conducted on admission showed a white blood cell count of 9.0 × 10^9^ L^−1^, with 81.5% polymorphonuclear leukocytes. The tumor markers were negative. A contrast-enhanced CT of the abdomen showed a clear invagination of the small intestine (Figs. 1 and 2). An administrative diagnosis of postoperative intestinal adhesion and intussusception was considered.

**Figure 3:**
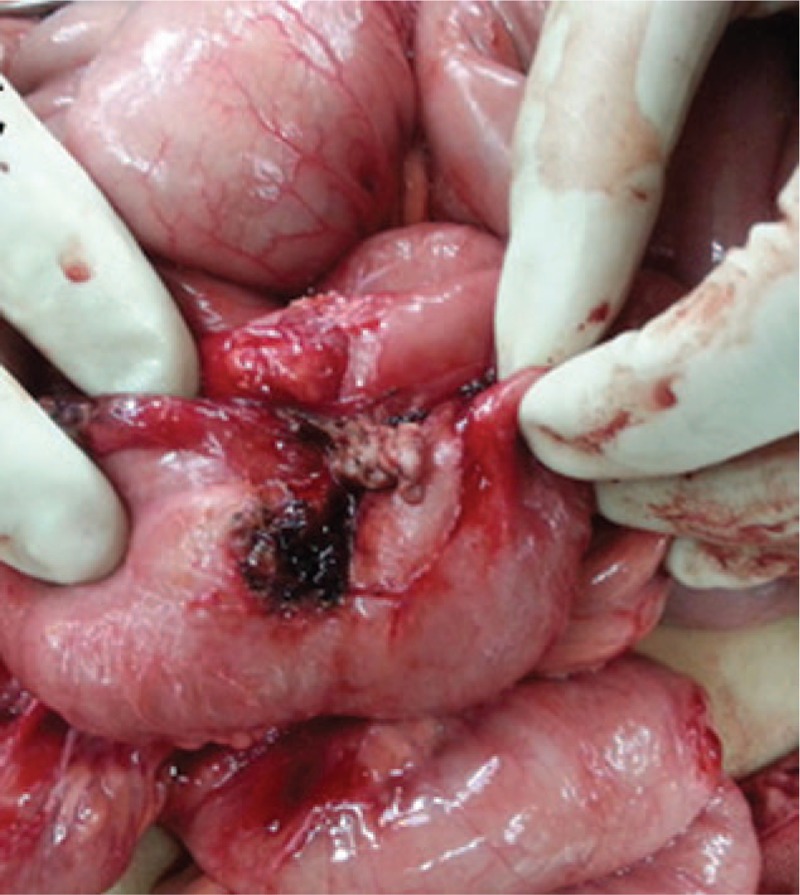
An adhesive mass of the ileum crossing the jejunum was found about 45 cm proximal to the ileocecal valve.

**Figure 4:**
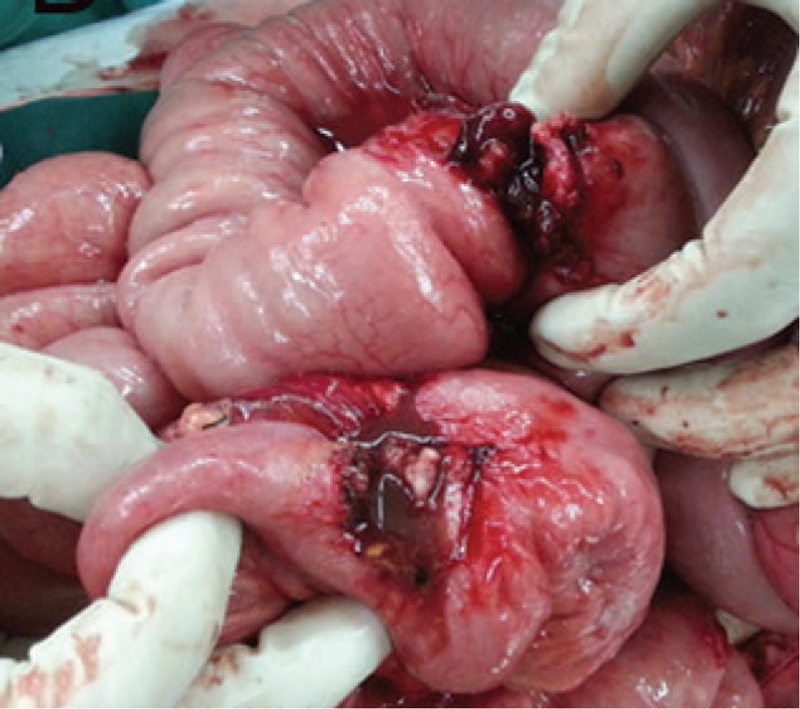
A jejunal-ileal cross-linked fistula was found in operation.

**Figure 5:**
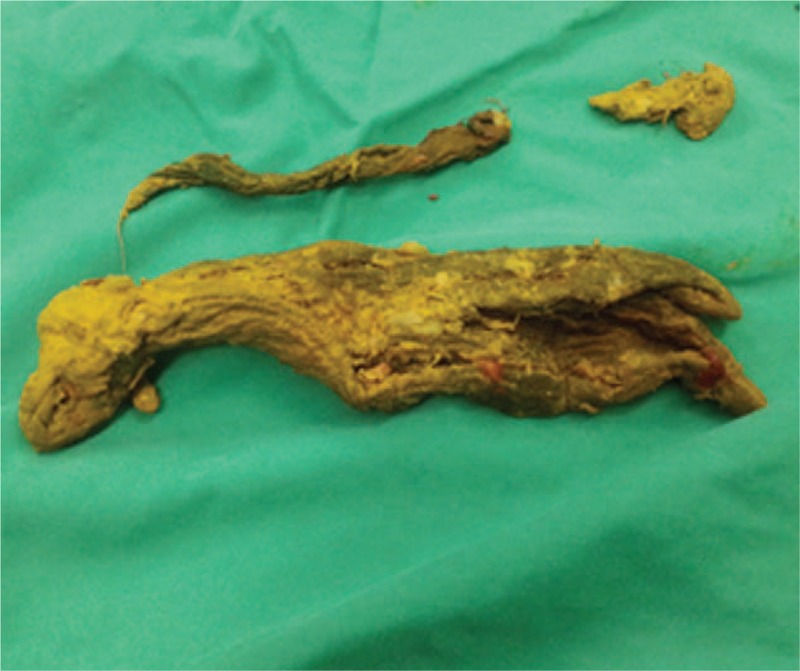
A RSS was found inside the ileal fistula. RSS = retained surgical sponge.

A laparotomy was performed. Intraoperatively, we found that the entire peritoneal cavity was very clean. An adhesive mass of the ileum crossing the jejunum was found about 45 cm proximal to the ileocecal valve (Fig. 3). This was consistent with the preoperative diagnosis of an intestinal adhesion. However, we could not find an intestinal segment with intussusception. After the adhesive intestine was detached, a jejunal-ileal cross-linked fistula was found (Fig. 4). More surprisingly, an RSS was found inside the ileal fistula when the cross-linked fistula was detached (Fig. 5). We then understood that the image of intussusception was a mimicking result of a complete transmural migration of the surgical sponge. We successfully removed the segments of intestine with the fistula and then made an anastomosis. Following a brief and uneventful postoperative period, the patient was discharged 7 days after surgery.

## 3 Discussion

To our knowledge, this is the first report showing an atypical image of a complete transmural migration of a RSS mimicking intussusception. RSS, usually referred to as GP, is an avoidable complication. Unfortunately, it still occurs. The reported incidence of GP varies from 1/1000 to 1/1500 for intra-abdominal operations.^[1]^ However, nonspecific symptoms and inclusive imaging still preclude an accurate diagnosis, especially in cases of complete transmural migration of RSSs. In the present case, calcified reticulate rind sign, an imaging feature typical of cases of complete transmural migration, is not observed. Instead, we identified an atypical image mimicking intussusception. By considering a patient's surgical history and the typical blood phenotype of upregulated polymorphonuclear leukocytes, surgeons may be able to improve their diagnostic accuracy.

Until now, only 4 cases of complete transmural migration of surgical sponges in humans have been reported. Various hypotheses have been formulated to explain exactly how the presumed transvisceral migration of such a foreign body might occur, and, moreover, without causing any particular parietal alteration. Dhillon and Park suggested that an inflammatory reaction surrounds the foreign body, and an abscess pouch forms and erodes the neighboring tissues.^[2]^ In a report on an animal experiment, Wattanasirichaigoon et al summarized 4 stages in the process of transmural migration of sponges: foreign body reaction, secondary infection, mass formation, and remodeling.^[3]^ Patil and his colleagues^[4]^ described that an increased pressure of the intra-abdominal mass on the bowel loops can lead to necrosis of the intestinal wall partially or entirely. Consequently, this process can lead to fistula or obstruction. The case we presented that an intraluminal surgical sponge was observed, which had completely migrated into the intestine without inducing peritoneal infection.
